# Avoiding anticoagulation drugs for postoperative atrial fibrillation enabled successful conservative treatment of left atrial dissection: a case report

**DOI:** 10.1186/s40792-021-01194-8

**Published:** 2021-05-12

**Authors:** Kentaro Kiryu, Takayuki Kadohama, Yoshinori Itagaki, Gembu Yamaura, Fuminobu Tanaka, Daichi Takagi, Takuya Wada, Itaru Igarashi, YuYa Yamazaki, Hiroshi Yamamoto

**Affiliations:** Department of Cardiovascular Surgery, Akita University Graduate School of Medicine, Hondo 1-1-1, Akita, 010-8543 Japan

**Keywords:** Left atrial dissection, Retrograde cardioplegia cannulation, Atrial fibrillation, Antiarrhythmic drugs, Anticoagulation

## Abstract

**Background:**

Left atrial dissection is a rare complication of cardiac surgery, most commonly associated with mitral valve surgery. Herein, we report on the successful conservative treatment of left atrial dissection while avoiding anticoagulation therapy.

**Case presentation:**

A 64-year-old man developed left atrial dissection during operation for acute type A aortic dissection, most likely due to retrograde cardioplegia cannulation. As there was no connection between the left atrial dissection cavity and the left atrium on enhanced computed tomography, we did not administer anticoagulants to prevent expansion of the left atrial dissection cavity. However, the patient developed atrial fibrillation, which was successfully managed by beta-blocker and amiodarone administration. Follow-up imaging showed gradual left atrial dissection reduction, and the patient was started on anticoagulation therapy.

**Conclusion:**

We were able to resolve left atrial dissection by preventing the use of anticoagulation therapy in the acute stage by managing the atrial fibrillation with antiarrhythmic drugs.

## Background

Left atrial dissection (LAD) is a rare complication of cardiac surgery. Its clinical presentation and course vary widely, from asymptomatic to severe, the latter being fatal due to hemodynamic collapse. Herein, we report on successful conservative treatment while avoiding anticoagulation therapy in a case of LAD, most likely related to retrograde cardioplegia cannulation, during operation for acute type A aortic dissection (ATAAD).

## Case presentation

**Fig. 1:**
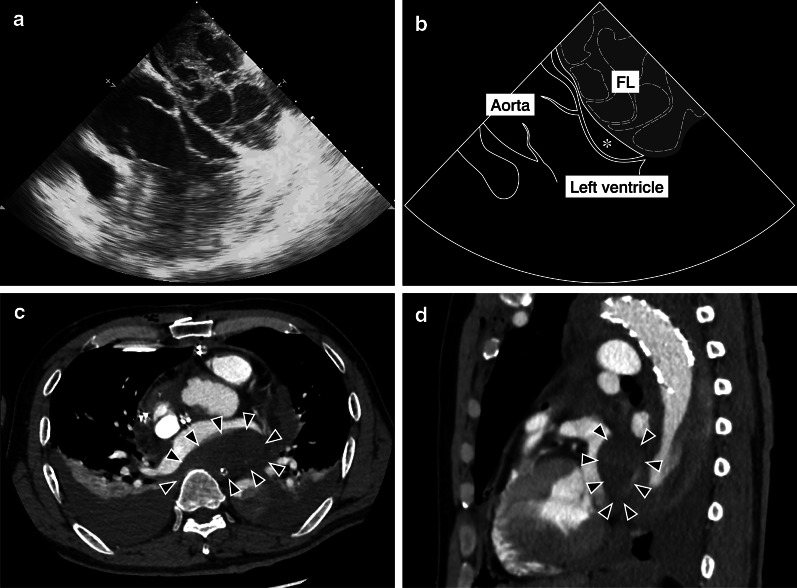
Intra- and postoperative imaging findings. **a** Transesophageal echocardiography showing a multivesicular space on the back of the left atrium that has no significant fistula with the left atrium. **b** A schema of the transesophageal echocardiography findings. The grayed area indicates false lumen (FL) of the left atrium. The true lumen of the left atrium (asterisk) is compressed by the FL. **c**, **d** Enhanced computed tomography (**c** axial view, **d** sagittal view) immediately after surgery showing a non-enhanced space on the back of the left atrium (arrowheads) that is not connected with the left atrium

A 64-year-old man was transferred to our hospital after being diagnosed with ATAAD. His vital signs were stable under administration of nicardipine and the echocardiography findings were unremarkable. We performed emergency total arch repair with frozen elephant trunk. Cardiopulmonary bypass (CPB) was established with bicaval drainage and left subclavian artery perfusion. We performed antegrade infusion for cardioplegia from the ascending aorta and retrograde infusion from the coronary sinus, inserting the cannula from the right atrial appendage. We always use a self-inflating retrograde cannula (RC014T^®^ 14Fr, Edwards Lifesciences Corp, Irvine, CA, USA). Cannula insertion for retrograde cardioplegia was smooth. The total cardioplegia dose was 20 ml/kg. The patient’s body weight was 70 kg; accordingly, the total cardioplegia dose was 1400 ml. The retrograde cardioplegia dose was one-third of the total dose. Upon infusion, the injection pressure rose to approximately 70 mm Hg, but it was momentary. We continued infusion after the pressure dropped to approximately 30 mm Hg and cardiac arrest was induced. During cardiac arrest, there were no signs of LAD, and the presence of retrograde cardioplegia solution was confirmed in the coronary orifices. At CPB weaning (after aortic cross de-clamping), transesophageal echocardiography (TEE) showed a multivesicular space on the back of the left atrium that had no significant fistula with the left atrium (Fig. 1a, b). As the patient’s vital signs were not affected, he was transferred to the intensive care unit after the operation. Follow-up TEE performed immediately after transfer to the intensive care unit again showed a space on the back of the left atrium, which at that time obstructed the left ventricle inflow. Enhanced computed tomography (CT) revealed a non-enhanced space on the back of the left atrium (Fig. 1c). We established the diagnosis of LAD. Because there was no connection between the LAD cavity and the left atrium on CT, we did not administer anticoagulant drugs to prevent expansion of the LAD cavity. However, the patient developed atrial fibrillation (AF) and his hemodynamics deteriorated. We considered that the LAD stimulated the left atrium and triggered AF. Therefore, we administered a beta-blocker (under intubation, landiolol hydrochloride was administered intravenously at 0–5 μg/kg/min, while after extubation, bisoprolol fumarate was given orally at a dose of 1.25–7.5 mg; the dose at discharge was 1.25 mg) and amiodarone, which successfully managed the AF, and the patient’s hemodynamics improved. During amiodarone administration, sinus rhythm was maintained.

**Fig. 2:**
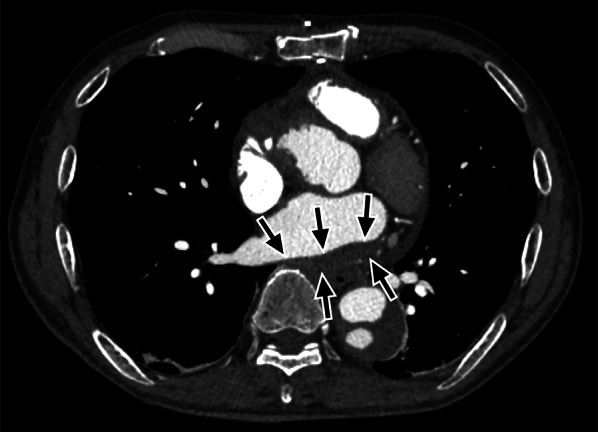
Follow-up imaging findings. Enhanced computed tomography on postoperative day 37 showing resolution of the left atrial dissection cavity (arrows)

The patient was extubated on postoperative day (POD) 5. Amiodarone administration was discontinued on POD 29, and he was started on anticoagulation therapy with a direct oral anticoagulant. We used warfarin potassium, with the dose adjusted so that the INR was approximately 2. There was residual paroxysmal AF rhythm, but the patient was hemodynamically stable. Follow-up enhanced CT performed on POD 37 showed LAD reduction (Fig. 2). The patient was discharged on POD 40.

## Discussion

LAD is a rare complication of cardiac surgery, associated with mitral valve surgery in 0.16–0.84% of cases [1, 2]. Tsukui et al. [1] reported a case of LAD related to retrograde cardioplegia cannulation, but they performed surgical treatment for the LAD. Other causes of LAD include insertion of left ventricular vent tube, aortic dissection, among others. In our case, the most likely cause was the retrograde cardioplegia cannulation. The patient was diagnosed with ATAAD, but enhanced CT before operation did not show LAD. Furthermore, there was no resistance or any problems at insertion of the left ventricular vent tube. The hemodynamic effects of LAD vary widely, from asymptomatic to hemodynamic collapse [2, 3]. TEE, CT, magnetic resonance imaging, and catheter study are helpful for LAD detection and diagnosis. In our case, enhanced CT was useful for LAD diagnosis. This was our first experience of LAD, and we could not diagnose it during the operation. However, intraoperative diagnosis of LAD is desirable. Regarding LAD treatment, according to the literature, reversal of anticoagulation could prevent LAD expansion [4]. In our case, follow-up CT demonstrated LAD resolution. However, in severe cases, surgical intervention should be considered. Currently, there are two types of surgical treatment: entry close and internal drainage [3]. In the available reviews [2–4], the postoperative mortality rate was 9.8–12.7%; also the mortality rate of medical treatment was 11.2–13.8%. In our case, conservative therapy was fortunately successful, but if worsening developed, we would have considered an internal drainage procedure. Furthermore, there was fortunately no occurrence of stroke. However, if the AF persisted, we intended to use unfractionated heparin. The use of heparin is debatable as it might contribute to LAD expansion. If we had to use unfractionated heparin, we would have adjusted the activated partial thromboplastin time at 30–45 s. Enhanced CT enabled us to diagnose LAD, which we considered to be related to the retrograde cardioplegia cannulation. The coronary sinus is a low-pressure system; it might prevent inflow to the LAD cavity and LAD expansion. In addition, the LAD cavity had no connection with the left atrium, which indicated the possibility of preventing LAD expansion. In a recent review by Cereda et al. [5], the authors recommended conservative care in cases of stable LAD. In our case, we avoided anticoagulation drug administration by managing the AF with antiarrhythmic drugs. Thus, we selected conservative treatment.

## Conclusion

In conclusion, it is important to correctly diagnose LAD and detect any connection with another cavity. In cases where no connection is detected, conservative treatment can be considered. In addition, preventing the use of anticoagulation drugs might help resolve LAD; in cases of postoperative AF, it is useful to administer antiarrhythmic drugs.

## Data Availability

Not applicable.

## Abbreviations

AF: Atrial fibrillation
ATAAD: Acute type A aortic dissection
CPB: Cardiopulmonary bypass
CT: Computed tomography
LAD: Left atrial dissection
POD: Postoperative day
TEE: Transesophageal echocardiography
